# Quality of surgical care in hospitals providing internship training in Kenya: a cross sectional survey.

**DOI:** 10.1111/tmi.12422

**Published:** 2014-11-19

**Authors:** Stephen Mwinga, Colette Kulohoma, Paul Mwaniki, Rachel Idowu, John Masasabi, Mike English, the SIRCLE Collaboration

**Affiliations:** ^1^ KEMRI‐Wellcome Trust Research ProgrammeNairobiKenya; ^2^ Ministry of HealthGovernment of KenyaNairobiKenya; ^3^ Vanderbilt Institute for Global HealthVanderbilt School of MedicineNashvilleTNUSA; ^4^ Nuffield Department of MedicineUniversity of OxfordOxfordUK

**Keywords:** quality of care, surgery, hospital, trauma, qualité des soins, chirurgie, hôpital, traumatisme

## Abstract

**Objective:**

To evaluate services in hospitals providing internship training to graduate doctors in Kenya.

**Methods:**

A survey of 22 internship training hospitals was conducted. Availability of key resources spanning infrastructure, personnel, equipment and drugs was assessed by observation. Outcomes and process of care for pre‐specified priority conditions (head injury, chest injury, fractures, burns and acute abdomen) were evaluated by auditing case records.

**Results:**

Each hospital had at least one consultant surgeon. Scheduled surgical outpatient clinics, major ward rounds and elective (half day) theatre lists were provided once per week in 91%, 55% and 9%, respectively. In all other hospitals, these were conducted twice weekly. Basic drugs were not always available (e.g. gentamicin, morphine and pethidine in 50%, injectable antistaphylococcal penicillins in 5% hospitals). Fewer than half of hospitals had all resources needed to provide oxygen. One hundred and forty‐five of 956 cases evaluated underwent operations under general or spinal anaesthesia. We found operation notes for 99% and anaesthetic records for 72%. Pre‐operatively measured vital signs were recorded in 80% of cases, and evidence of consent to operation was found in 78%. Blood loss was documented in only one case and sponge and instrument counts in 7%.

**Conclusions:**

Evaluation of surgical services would be improved by development and dissemination of clear standards of care. This survey suggests that internship hospitals may be poorly equipped and documented care suggests inadequacies in quality and training.

## Abstract (fr)

**Objectif:**

Evaluer les services dans les hôpitaux offrant des stages de formation à des médecins diplômés au Kenya.

**Méthodes:**

Enquête auprès de 22 hôpitaux offrant des stages de formation. La disponibilité des ressources clés incluant infrastructure, personnel, matériel et médicaments a été évaluée par observation. Les résultats et processus de soins pour des affections prioritaires prédéfinies (blessure à la tête, blessure à la poitrine, fractures, brûlures et maux d'estomac aigus) ont été évalués par l'audit des dossiers des cas.

**Résultats:**

Chaque hôpital avait au moins un chirurgien consultant. Les cliniques chirurgicales ambulatoires planifiées, les principales tournées dans les chambres d'hospitalisation et des listes d'opérations choisies (demi‐journée) ont été fournies une fois par semaine dans 91%, 55% et 9% des cas respectivement. Dans tous les autres hôpitaux, cela a été effectué deux fois par semaine. Les médicaments de base n’étaient pas toujours disponibles (par ex. la gentamicine, la morphine et la péthidine dans 50% des hôpitaux, les pénicillines anti‐staphylococciques injectables dans 5%). Moins de la moitié des hôpitaux disposaient de toutes les ressources nécessaires pour fournir de l'oxygène. 145 sur 956 cas évalués ont subi des opérations sous anesthésie générale ou rachidienne. Nous avons retrouvé des notes d'opération pour 99% des cas et des dossiers d'anesthésie pour 72%. Les mesures préopératoires des signes vitaux ont été enregistrées dans 80% des cas et la preuve du consentement pour l'opération a été trouvée dans 78% des cas. La perte de sang a été documentée dans un seul cas et le comptage des éponges et instruments dans 7% des cas.

**Conclusions:**

L’évaluation des services de chirurgie serait améliorée par le développement et la dissémination de normes de soins claires. Cette étude suggère que les hôpitaux offrant des stages peuvent être mal équipés et les soins enregistrés suggèrent des insuffisances dans la qualité et la formation.

## Abstract (es)

**Objetivo:**

Evaluar los servicios en hospitales que proveen entrenamiento a médicos graduados en Kenia.

**Métodos:**

Estudio en 22 hospitales universitarios con entrenamiento de médicos residentes. Se evaluó mediante observación la disponibilidad de recursos claves, incluyendo infraestructura, personal, equipamiento y medicamentos. Se evaluaron los resultados y procesos de cuidados para condiciones prioritarias especificadas previamente (traumatismo craneoencefálico, lesión torácica, fracturas, quemaduras y abdomen agudo) mediante la auditoría de historias clínicas.

**Resultados:**

Cada hospital tenía al menos un cirujano consultor. Se entregaban una vez por semana las listas de intervenciones quirúrgicas programadas en clínicas ambulatorias, en las rondas de visitas a las principales salas y las cirugías electivas (medio día) en 91%, 55% y 9% de los hospitales, respectivamente. En los demás hospitales se llevaban a cabo dos veces por semana. Los medicamentos básicos no estaban siempre disponibles (ej. gentamicina, morfina y meperidina en 50%, penicilina anti‐estafilocócica inyectable 5% de los hospitales). Menos de la mitad de los hospitales tenían todos los recursos necesarios para proveer oxígeno. En 145 de 956 casos evaluados se llevó a cabo la cirugía con anestesia general o intradural. Encontramos apuntes quirúrgicos para un 99% y registros de la anestesia para el 72%. Se tenían registros de los signos vitales pre‐quirúrgicos en un 80% de los casos y evidencia del consentimiento del paciente a ser intervenido en un 78%. Se documentaba pérdida de sangre solo en un caso y conteo de esponjas e instrumental en un 7%.

**Conclusiones:**

La evaluación de los servicios quirúrgicos podría mejorarse mediante el desarrollo y la diseminación de estándares de cuidados precisos. Este estudio sugiere que los hospitales universitarios podrían estar mal equipados y los cuidados documentados sugieren que existen deficiencias tanto a nivel calidad como en el entrenamiento.

## Introduction

In many countries, health systems are organised on the basis of a district or equivalent administrative unit. These typically contain a district hospital linked to a network of primary care and community‐based health services. From a systems perspective, and as recognised since Alma Ata (WHO 1987), it is critical that district hospitals deliver high‐quality care effectively and efficiently. Hospitals should allow this through delivery of more complex forms of health care in a setting that concentrates skills and resources (English *et al*. 2006). In the case of surgery, there is some evidence that provision of basic surgical services can be highly cost‐effective in low‐ and middle‐income settings (McCord & Chowdhury 2003) and such services are likely to gain in importance in settings such as Kenya, as life expectancy increases and trauma and non‐communicable diseases grow in importance (Lozano *et al*. 2012).

However, reports spanning maternal and paediatric care suggest that hospitals often fail to deliver good services in low‐income settings, but there are few reports on surgical services (Nolan *et al*. 2000; English *et al*. 2004; Kingham *et al*. 2009; Kruk *et al*. 2010). Donabedian (1978) described the most widely used simple framework for examining healthcare delivery, dividing it into three major aspects: structure (the nature of the inputs available to provide care), process (the actions taken to convert inputs into the care provided) and outputs or outcomes (the consequences of care). The latter are not limited to health status but may encompass satisfaction, costs or other valued outputs depending on one's perspective. Unfortunately, routine health information systems (HIS) in low‐income countries (LIC) are often unable to provide reliable, disease‐specific outcome data, and routine data based on process indicators are also lacking (Kihuba *et al*. 2014). In the absence of routine data, a survey approach was designed to assess the quality of routine surgical services in Kenyan hospitals in collaboration with the Ministry of Health.

## Methods

### Selection of indicators

There were no nationally approved quality indicators for surgical services provided in hospitals in Kenya prior to this survey. National guidelines spanning common surgical presentations had, however, been published in 2009 by the Ministry of Health. Performance indicators were therefore developed with the Ministry of Health using these and WHO resources as reference standards (Ministry of Health Republic of Kenya 2009; WHO 2013). Initially, this team prioritised assessment of five specific admission conditions: head injury, chest injury, long‐bone fracture, burns and acute abdomen. Indicators spanned availability of essential equipment and resources (structure) and provision of care in three domains – assessment of key elements of the admission history and examination, general management (including investigation and adjunctive medical or supportive therapy) and operative care – were undertaken. Individual indicators were constructed to allow an ‘achieved’ (1) or ‘not achieved’ (0) assessment based on direct observation for facility‐level structure indicators or examination of case records for process of care indicators. The latter has the limitation of evaluation based only on what is documented about the care provided but the advantage of making examination of multiple patient events possible within reasonable timescales. Draft tools were pilot‐tested in a hospital not included in the survey with revisions made for clarity as required, and a detailed standard operating procedure was developed to guide its use.

### Selection of hospitals and sample size

Kenya has 235 public hospitals that should be able to offer first referral‐level surgical services provided by general medical officers (e.g. non‐operative fracture management, minor surgery) as a minimum standard of care. Of these, 40 are ‘internship training centres’ with (i) consultants in each of the four major inpatient disciplines (surgery, internal medicine, obstetrics and paediatrics) and (ii) basic laboratory, pharmacy, radiology services and at least one working theatre. Such centres provide supervised training to graduate clinical staff during a 1‐year compulsory internship period prior to full registration. These provide both routine and emergency operative care for major cases with the support of physician or non‐physician assistant anaesthesiologists. Young clinicians leaving these internship hospitals then often lead service delivery in smaller hospitals.

Study hospitals (*n* = 22) were purposely selected by the Ministry to provide a logistically feasible and geographically representative sample of the 40 internship hospitals. With 22 hospitals to be assessed, we explored how precisely we might report estimates of compliance with quality indicators. Taking hospitals as the units of clustering, assuming a design effect of 1.5 based on a previous study in district hospitals (Ayieko *et al*. 2011) and anticipating reporting 50% or 10% correct performance, we estimated that a minimum of 12 and four cases per hospital featuring the included diagnoses listed above would be required to report estimates with a precision of ±7.5% (95% CI). The case records included in the survey were identified from ward registers by working backwards from 31 May 2012 (just prior to survey visit) until 15 cases of a specific diagnosis most proximate to the survey were retrieved or the time available for survey work was exhausted.

### Survey conduct

Data collection was performed over 5 weeks in June and July 2012. Survey staff comprised 22 Ministry employees (nurses, records officers or clinical officers) with one drawn from each hospital selected for survey. All staff underwent 1 week of training together that included a pilot survey in a non‐study hospital. Staff were subsequently divided into five teams (4–5 per team) that each visited 4–5 hospitals for 3–4 days during which they also undertook other survey activities. Each team was led by a researcher who was also an experienced health worker. Resource availability (structure) was assessed by team leaders, who conducted a facility walk‐through using a standard checklist. Process of care and outcome data were entered directly into laptops from the case records using REDCap^®^ (Research Electronic Data Capture) (Harris *et al*. 2009). Additional source documents included prescription records, monitoring and discharge charts and operative notes where applicable.

Real‐time range and consistency checks were run as the data were entered. At the end of each data collection day, data entries were examined for errors using procedures executed in STATA^®^ v10 (Stata Corps, TX, USA). Corrections were ultimately made by referring back to the source document under the supervision of the team leader. The clean data files from all study hospitals were then uploaded into a central REDCap server daily for secure backup of study data.

### Analysis

Availability of essential resources was scored using dichotomous indicators (0: absent *vs*. 1: present) for each structure item for each hospital. The simple proportion of the 22 hospitals complying with each indicator could then be reported. For process indicators, with multiple cases assessed in each hospital, we calculated the proportions of cases complying with the quality indicator together with 95% confidence intervals adjusted for clustering within hospitals. In a number of areas, such as documentation of elements of the primary and secondary survey, dichotomous indicators were summed to derive a score with a denominator equal to the number of tasks. Such scores have the limitation of focusing on what is documented as an indication of what is done that is common to most clinical audit. For these scores, we began by calculating the median score for all cases within a hospital and then report the median of the 22 hospitals’ scores together with the interquartile range.

### Ethical approval

Scientific and ethical approval was obtained from the KEMRI Ethical Review Committee. The Ministry of Health provided administrative permission, and assent was obtained from the hospital management prior to surveys.

## Results

Surveys were successfully conducted in the 22 hospitals approached with all data collected in a period of 5 weeks in June and July 2012.

### Availability of basic resources

All hospitals had a consultant surgeon [22/22 (100%)]. Separate surgical wards were available in 18/22 (82%). In 4/22 (18%) hospitals, surgical patients were cared for on a joint medical/surgical ward. Special wards for burns and orthopaedic and paediatric cases were available in 3/22 (14%), 7/22 (32%) and 4/22 (18%) hospitals, respectively. We observed surgical patients sharing beds in 4/22 (18%) hospitals. Consultants provided surgical outpatient clinics for booked cases, led major surgical ward rounds and ran elective (half day) theatre lists once per week in 20/22 (91%), 12/22 (55%) and 2/22 (9%) hospitals, respectively. In all other hospitals, these scheduled clinics, major rounds or theatre lists were conducted twice per week. Emergency theatre cases or lists were scheduled as needed and feasible with cases often handled by post‐internship general medical officers.

On the day of survey, consistent running water was available on wards in 19/22 (86%) hospitals and soap in 16/22 (73%). Only 7/22 (32%) had alcohol hand rubs. There were discarded sharps overflowing from containers in 6/22 (27%) hospitals. National clinical guidelines for common surgical problems were available in 3/22 (14%) hospitals and wall charts for management of surgical emergencies in 5/22 (23%). Clinical audits and regular staff meetings were reported in 12/22 (55%) hospitals, although only 8/22 (36%) had minutes for these meetings available for review at the time of the survey.

Crystalline penicillin was available in all hospitals. Gentamicin, amoxicillin, oral antistaphylococcal penicillins and ciprofloxacin were available in half of study hospitals (11/22, 50%), but injectable antistaphylococcal penicillins were available in only one hospital (5%). Ceftriaxone and chloramphenicol were available in 9/22 (41%) hospitals. Analgesics had better availability. Diclofenac and paracetamol were available in almost all facilities. Morphine and pethidine were present in half of the hospitals (11/22, 50%). Heparin and cimetidine were available in fewer than half of the hospitals (7/22, 32%). With regard to anaesthetic agents, bupivacaine for spinal blocks was available in 5/22 (23%) hospitals only, but all hospitals had inhalational agents. All hospitals had nasogastric tubes. Intravenous fluid administration sets, gloves (both clean and sterile) and urethral catheters were available in at least three quarters of hospitals. Fewer than half of hospitals had both a source of oxygen (typically cylinders) and a working flowmeter on the surgical ward.

### Process

Overall 956 case records were reviewed. Among these selected records, the diagnoses were distributed as follows: head injury *n = *178 (19%), chest injury *n = *136 (14%), acute abdomen *n = *220 (23%), long‐bone fractures *n = *237 (25%) and burns *n = *185 (19%). A summary of patient characteristics is provided in Table 1. The sampling approach does not allow reliable estimation of true prevalence. Identification of chest injury diagnoses was most difficult as this event was rare. Irrespective of diagnosis, more than 80% of cases presented directly to the hospital from home or the site of trauma rather than being referred from another facility. Key demographic data were missing in from 5% to 17% cases (detailed in Table 1). Where available, included case records indicated a preponderance of male cases for trauma‐related admissions [specifically head injury, chest injury and fractures (81%, 86% and 71% respectively, detailed in Table 1)]. Additionally, the study population was young with the median age for cases reviewed <40 years for all conditions, and for those with burns, the median age of cases was only 7 years. Overall, mortality across all cases examined was low (1.9%) with the highest fatality in the group of cases of acute abdomen (4.3%, detailed in Table 1). Referrals to a higher level of care were most frequent (25/178, 14%) for head‐injured patients. Time of admission was recorded in the case records on fewer than 30% of reviewed records (detailed in Table 1), regardless of the diagnosis. Similarly temperature, respiratory rate, pulse and blood pressure were recorded in the clinical records of, respectively, fewer than 40%, 31%, 36% and 30% of admissions, regardless of the diagnosis (detailed in Table 1).

**Table 1:** Characteristics of admitted patients for whom case records were retrieved and care evaluated

Characteristic	Head injury	Chest injury	Acute abdomen	Long‐bone fractures	Burns	Overall
	*N* = 178	*N* = 136	*N* = 220	*N* = 237	*N* = 185	*N* = 956
Referrals	28/178 (15.7) [10.2–23.4]	15/136 (11.0) [6.2–19.0]	31/220 (14.1) [8.2–23.1]	31/237 (13.1) [8.2–20.2]	19/185 (10.3) [5.8–17.5]	124/956 (13.0) [9.0–18.4]
Date of admission recorded	176/178 (98.9) [95.4–99.7]	130/136 (95.6) [82.7–99.0]	216/220 (98.2) [95.0–99.3]	228/237 (96.2) [90.1–98.6]	178/185 (96.2) [88.7–98.8]	928/956 (97.1) [91.8–99.0]
Sex (missing)	17/178 (9.6) [3.9–21.4]	9/136 (6.6) [2.1–19.0]	17/220 (7.7) [3.1–18.1]	17/237 (7.2) [2.7–17.8]	23/185 (12.4) [5.6–25.5]	83/956 (8.7) [4.0–18.0]
Sex (male)	131/161 (81.4) [68.8–89.6]	109/127 (85.8) [73.2–93.1]	125/203 (61.6) [48.0–73.5]	155/220 (70.5) [57.4–80.8]	88/162 (54.3) [42.8–65.4]	608/873 (69.6) [61.3–76.9]
Age categories (years)						
Missing	30/178 (16.9) [8.3–31.2]	23/136 (16.9) [6.9–36.0]	25/220 (11.4) [5.3–22.6]	31/237 (13.1) [5.4–28.4]	22/185 (11.9) [5.6–23.6]	131/956 (13.7) [6.8–25.6]
≤12.9	20/148 (13.5) [7.5–23.1]	3/113 (2.7) [0.9–7.3]	10/195 (5.1) [2.4–10.5]	24/206 (11.7) [6.6–19.7]	94/163 (57.7) [43.8–70.4]	151/825 (18.3) [13.4–24.4]
13–45.9	110/148 (74.3) [64.7–82.0]	81/113 (71.7) [63.6–78.6]	147/195 (75.4) [66.4–82.6]	109/206 (52.9) [43.5–62.1]	58/163 (35.6) [24.4–48.6]	505/825 (61.2) [55.6–66.5]
≥46	18/148 (12.2) [7.4–19.4]	29/113 (25.7) [18.3–34.7]	38/195 (19.5) [13.6–27.1]	73/206 (35.4) [27.2–44.7]	11/163 (6.7) [3.4–13.0]	169/825 (20.5) [17.1–24.4]
Age: median (IQR)	26.0 (20.0–36.5)	34.0 (25.0–46.0)	28.0 (22.0–41.0)	36.0 (25.0–54.0)	7.0 (2.0–27.0)	28.0 (19.0–41.0)
Outcome						
Missing	11/178 (6.2) [2.5–14.6]	8/136 (5.9) [2.4–13.9]	11/220 (5.0) [2.7–9.1]	15/237 (6.3) [2.9–13.4]	11/185 (5.9) [2.7–12.7]	56/956 (5.9) [3.1–10.7]
Discharged	136/167 (81.4) [72.3–88.0]	114/128 (89.1) [79.8–94.4]	185/209 (88.5) [80.0–93.7]	195/222 (87.8) [75.2–94.5]	169/174 (97.1) [93.3–98.8]	799/900 (88.8) [83.6–92.4]
Absconded	2/167 (1.2) [0.3–4.9]	1/128 (0.8) [0.1–5.9]	1/209 (0.5) [0.1–3.6]	0/222 (0.0) [0.0]	1/174 (0.6) [0.1–4.7]	5/900 (0.6) [0.2–1.3]
Transferred/ referred	25/167 (15.0) [8.2–25.7]	12/128 (9.4) [4.5–18.6]	14/209 (6.7) [3.7–11.9]	27/222 (12.2) [5.5–24.8]	1/174 (0.6) [0.1–4.7]	79/900 (8.8) [5.5–13.8]
Dead	4/167 (2.4) [0.7–7.5]	1/128 (0.8) [0.1–6.2]	9/209 (4.3) [1.7–10.3]	0/222 (0.0) [0.0]	3/174 (1.7) [0.6–5.2]	17/900 (1.9) [0.9–3.9]
Time of admission recorded	47/178 (26.4) [13.9–44.4]	29/136 (21.3) [12.7–33.5]	41/220 (18.6) [9.9–32.4]	50/237 (21.1) [10.1–38.9]	41/185 (22.2) [10.2–41.6]	208/956 (21.8) [11.7–36.9]
Temperature recorded on admission	51/178 (28.7) [18.8–41.1]	40/135 (29.6) [16.0–48.3]	85/219 (38.8) [25.9–53.5]	73/237 (30.8) [19.0–45.8]	54/185 (29.2) [16.2–46.7]	303/954 (31.8) [21.5–44.2]
Clinician take and record respiratory rate	54/178 (30.3) [17.4–47.5]	35/135 (25.9) [12.8–45.6]	60/219 (27.4) [14.8–45.1]	60/236 (25.4) [14.1–41.5]	29/185 (15.7) [8.0–28.4]	238/953 (25.0) [14.6–39.2]
Clinician take and record pulse	63/178 (35.4) [21.3–52.6]	39/135 (28.9) [15.5–47.3]	71/219 (32.4) [19.1–49.3]	58/237 (24.5) [13.4–40.5]	32/185 (17.3) [9.0–30.7]	263/954 (27.6) [17.1–41.3]
Clinician take and record blood pressure	53/178 (29.8) [19.9–42.0]	39/135 (28.9) [17.2–44.3]	64/219 (29.2) [20.3–40.1]	54/237 (22.8) [13.7–35.5]	13/185 (7.0) [3.9–12.3]	223/954 (23.4) [16.9–31.4]
Booked for theatre at admission	6/178 (3.4) [1.1–10.0]	11/135 (8.1) [3.7–17.0]	66/219 (30.1) [19.9–42.8]	50/237 (21.1) [15.2–28.6]	10/185 (5.4) [2.8–10.3]	143/954 (15.0) [11.1–19.9]
Patient taken to theatre	6/178 (3.4) [0.9–11.5]	9/135 (6.7) [3.1–13.6]	84/220 (38.2) [27.3–50.5]	55/237 (23.2) [17.6–29.9]	17/185 (9.2) [5.5–15.0]	171/955 (17.9) [13.8–22.9]

Results presented include % in parentheses and 95% confidence intervals adjusted for clustering at hospital level in square brackets unless otherwise specified.

### Selected diagnoses: trauma

Road traffic accidents were responsible for 53%, 40% and 42% admissions with head injury, chest injury and fracture, respectively (see Table 2 for detail). Table 2 also provides values for the number of multiple‐injury patients. We examined records for documentation of a primary survey evaluating airway, breathing and circulation at admission for each of our included trauma diagnoses (Table 2). A secondary survey comprises a fuller assessment of seven key areas (for detail, see Table 2). The median number of secondary survey assessment tasks documented in trauma patients’ case records was very low, at 2/7, 1/7 and 1/7 for head injury, chest injury and fracture diagnoses, respectively. Rather than documenting a full secondary survey, these findings suggest that clinicians focused, at least for documentation, on areas anticipated to be closely related to the primary cause of admission. For example, examination of the head and face (65%) and a coma scale assessment or pupillary responses (42%) were documented in head‐injured patients, and chest examination (65%) and extremity examination (65%) were documented in chest injury and fracture patients, respectively (see Table 2 for detail). When further detailed information on the injuries was sought from records, this was rarely found. For example, assessments of peripheral pulses or for compartment syndrome were documented in fewer than 19/237 (8%) long‐bone fracture cases, while auscultation for bilateral breath sounds and palpation for chest wall crepitus were documented in fewer than 34/136 (25%) chest injury cases.

**Table 2:** Characteristics and performance against indicators for admissions with injuries

Characteristic/Indicator	Head injury	Chest injury	Long‐bone fractures
	*N* = 178	*N* = 136	*N* = 237
Mechanism of injury categories			
Missing	1/178 (0.6) [0.1–4.3]	2/136 (1.5) [0.4–5.4]	3/237 (1.3) [0.4–4.2]
Assault	61/177 (34.5) [26.8–43.0]	55/134 (41.0) [29.9–53.2]	26/234 (11.1) [8.1–15.1]
Road traffic accidents	93/177 (52.5) [42.9–62.0]	54/134 (40.3) [29.5–52.1]	99/234 (42.3) [34.4–50.6]
Other	23/177 (13.0) [7.9–20.7]	25/134 (18.7) [12.6–26.7]	109/234 (46.6) [38.6–54.7]
Multiple injuries	44/177 (24.9) [16.4–35.7]	34/134 (25.4) [14.8–39.9]	31/236 (13.1) [8.1–20.6]
Components of primary examination documented			
Airway	51/178 (28.7) [18.3–41.9]	52/136 (38.2) [21.5–58.3]	28/237 (11.8) [5.4–23.9]
Breathing	44/178 (24.7) [14.3–39.3]	48/136 (35.3) [19.9–54.5]	30/237 (12.7) [6.2–24.3]
Circulation	18/178 (10.1) [5.2–18.9]	24/136 (17.6) [8.5–33.2]	24/237 (10.1) [4.5–21.1]
Primary examination score – range, median (IQR)	0–3, 0 (0–1)	0–3, 0 (0–2)	0–3, 0 (0–0)
Primary examination score as percentages – range, median (IQR)	0–100, 0 (0–33)	0–100, 0 (0–67)	0–100, 0 (0–0)
Components of secondary examination documented			
Head and face (incl. eyes and ears)	116/178 (65.2) [46.9–79.8]	23/136 (16.9) [8.9–29.7]	15/237 (6.3) [2.8–13.8]
Neck	21/178 (11.8) [4.8–26.2]	20/136 (14.7) [7.6–26.6]	9/237 (3.8) [1.6–8.6]
Chest	32/178 (18.0) [10.2–29.8]	88/136 (64.7) [42.4–82.0]	16/237 (6.8) [3.8–11.6]
Abdomen	19/178 (10.7) [5.4–20.1]	15/136 (11.0) [5.7–20.1]	10/237 (4.2) [1.9–9.1]
Back (incl. spine and rectal examination)	7/178 (3.9) [1.9–7.9]	7/136 (5.1) [2.2–11.5]	3/237 (1.3) [0.3–5.3]
Extremities	24/178 (13.5) [8.1–21.7]	10/136 (7.4) [2.8–18.0]	155/237 (65.4) [43.0–82.6]
Neurologic (pupils, Glasgow Coma Scale)	74/178 (41.6) [27.1–57.6]	11/136 (8.1) [4.0–15.7]	11/237 (4.6) [1.7–11.9]
Secondary examination score – range, median (IQR)	0–7, 2 (1–2)	0–7, 1 (0–2)	0–6, 1 (0–1)

Results presented include % in parentheses and 95% confidence intervals adjusted for clustering at hospital level in square brackets unless otherwise specified.

### Burns

Documentation of the total body surface area (%TBSA) was missing in 25/185 (14%) of burn cases but when recorded was mostly <20% (Table 2). Most (154/185, 83%) burn cases were prescribed antibiotics, most commonly flucloxacillin (88/154, 57%), benzylpenicillin (37/154, 24%) or metronidazole (34/154, 22%). Topical antiseptics (101/185, 55%) or ointments (114/185, 62%) were also commonly used, while surgical debridement (19/185, 10%) or skin graft (2/185, 1%) was rare. Intravenous fluids were used in 82/185 (44%) cases with a crystalloid containing approximately physiological sodium concentrations used in 69/82 (84%) cases and 5% dextrose used in 13/82 (16%). In only 16/82 (20%) cases, body weight was recorded and used in the fluid plan calculation.

### Acute abdomen

Symptoms commonly identified in patients with acute abdomen were variably documented as follows: vomiting (178/220, 81%), passage of flatus (107/220, 49%) and passage of melaena stool (24/220, 11%). The same was true for signs: tenderness (182/220, 83%), guarding (94/220, 43%) and rebound tenderness (78/220, 35%). Half (111/220, 50%) cases had a complete blood count performed at admission, but very few (9/220, 4%) had a bedside urine test and only 1/220 (<1%) had either urine or blood cultured. However, 152/220 (69%) were started on intravenous fluids and 171/220 (78%) on parenteral antibiotics [metronidazole (127/171, 74%) and ceftriaxone (78/171, 46%)]. Ultimately, 84/220 (38%) cases had surgery (see below) with 66/84 (79%) of these planned from admission (Table 1).

### Surgical procedures

A total of 171/956 (18%) admissions were documented as taken to theatre for surgical procedures. This was uncommon for head‐injured patients (6/178, 3%) and most frequent for those admitted with acute abdomen (84/220, 38%) (Table 1). The form of anaesthesia used was not documented in 12/171 (7%) cases. Where documented, general anaesthesia was employed in 128/159 (81%), spinal anaesthesia in 17/159 (11%) and other forms of sedation or local anaesthesia in the remainder. Assuming that cases in which general or spinal anaesthesia was used to represent more major cases (*n *=* *145), we examined how well the surgical procedures were documented, focusing on elements that might be considered universal standards of care for professionals or linked to patient safety (Table 3). Of these 145 major cases, we found operation notes in the record for 143 (99%). There was a clear indication of who the surgeon was for 130/145 (90%) cases but far fewer contained a formally signed record of surgery (94/145, 65%). Anaesthetic records were less commonly found (104/145, 72%), and the identity of the anaesthetist could be determined in half of cases with spinal or general anaesthesia (72/145, 50%). Pre‐operatively measured vital signs of pulse, blood pressure and temperature were noted in approximately 116/145 (80%) cases (Table 3), but time of last meal (56/145, 39%) and any pre‐medication (33/145, 23%) were less commonly recorded. Evidence of consent to operation was found in 113/145 (78%) cases. Records of the operation reported findings in 120/145 (83%) cases and the nature of the procedure in 137/145 (95%) and documented the patient's condition at the end of the surgery (either in surgical or in anaesthetic notes), the drugs used for induction or maintenance of anaesthesia and vital signs during the operative procedure in two‐thirds (Table 3). Blood loss was documented in only one case and sponge and instrument counts in 7% (10/145).

**Table 3:** Characteristics and performance against indicators for admissions undergoing surgery requiring spinal or general anaesthesia

Indicators	
Time of last meal documented	56/145 (38.6) [20.7–60.2]
Operation notes written	143/145 (98.6) [94.5–99.7]
Operation notes signed	94/145 (65.0) [50.6–78.2]
Surgeon's name written in operation notes	130/145 (90.1) [78.9–96.4]
Anaesthetist complete anaesthetic record after procedure done	104/145 (71.7) [56.2–83.4]
Anaesthetist name identifiable	72/145 (49.7) [31.7–67.7]
Records with documentation of items of pre‐operative checklist	
Consent	113/145 (77.9) [62.0–88.4]
BP	117/145 (80.7) [63.8–90.8]
Temperature	119/145 (82.1) [64.0–92.2]
Pulse rate	119/145 (82.1) [63.9–92.2]
Pre‐medication	33/145 (22.8) [7.3–52.3]
Records with documentation by surgeon of items relevant to operative procedure	
Patient name	134/145 (92.4) [83.0–96.8]
IP number	106/145 (73.1) [51.3–87.5]
Findings during operation	120/145 (82.8) [66.9–91.9]
Procedure performed	137/145 (94.5) [86.4–97.9]
Estimated blood loss	1/145 (0.7) [0.1–5.4]
Verification of sponge/instrument count	10/145 (6.9) [2.7–16.7]
Patient's clinical status at conclusion of operation	97/145 (66.9) [49.9–80.4]
Records with documentation of items by anaesthesiologist relevant to anaesthetic procedure	
Patient name	102/145 (70.3) [54.7–82.3]
IP number	86/145 (59.3) [41.7–74.8]
Medications used during induction of anaesthesia	98/145 (67.6) [53.3–79.2]
Medications used during procedure	101/145 (69.7) [54.2–81.7]
Vital signs	96/145 (66.2) [53.0–77.3]
Patient's clinical status at conclusion of operation	78/145 (53.8) [37.6–69.2]

Results presented include % in parentheses and 95% confidence intervals adjusted for clustering at hospital level in square brackets unless otherwise specified.

## Discussion

Quality and safety of surgical care have been of concern in developed countries for many years driven by desires to improve patient outcomes, to reduce health system costs attributable to poor care and, perhaps most powerfully in some countries, to avert litigation (Maggard‐Gibbons 2014). Considerable efforts aimed at improvement span highly structured ways of working, often guided by checklists (Soar *et al*. 2009), and professional and public scrutiny of performance (Maggard‐Gibbons 2014). In low‐income settings, we know little about the challenges facing surgeons in non‐tertiary hospital settings and little about the quality of services or patient outcomes. Published research suggests major deficiencies (Kingham *et al*. 2009; Notrica *et al*. 2011; Groen *et al*. 2012). In this study, we evaluated the quality of surgical services in hospitals providing experiential (internship) training to young doctors. These hospitals had a qualified surgeon who had undergone 3 years of postgraduate specialist training and might be expected to offer a higher technical level of care than the majority of Kenya's public hospitals. It is therefore of concern that basic resources (including running water, soap and first‐line drugs) were not uniformly available and that surgical patients had to share beds.

Assessing quality of the process of care typically requires that standards or criteria are defined and disseminated. Explicit standards for basic surgical services have not been developed in Kenya. National guidance in narrative form in books aimed at practitioners was developed by the Ministry of Health 3 years prior to this study (Ministry of Health Republic of Kenya 2009), but these or other practice guidelines were found in very few hospitals. The default position is therefore that surgeons within the hospitals set standards through example and supervision. Formal opportunities for this form of oversight typically seemed limited to scheduled outpatient clinics, ward rounds and half‐day sessions allocated to conduct of elective operations; all typically conducted once or at most twice per week. The contribution surgeons make to emergency care or other forms of training and practice was not evaluated in this study, but anecdotal reports suggest that these are often limited.

To address the gap in explicit standards, we worked with Ministry of Health staff to identify basic indicators for this survey drawing on local knowledge, professional norms and existing WHO tools (WHO 2013). Because policy makers requested that specific causes of surgical admission be prioritised for study, we cannot provide information on the general pattern of surgical admissi‐ons to hospitals. Amongst the 956 cases studied, three quarters (Table 1) were likely the result of trauma or accidents (head injury, chest injury, fractures and burns) and most (80%) presented directly to the hospital. We noted major deficiencies in documentation of the findings of primary and secondary surveys in these patients. We acknowledge that documentation is an imperfect measure of whether the examination was actually performed, but Kenyan policy makers deemed such documentation good practice, a sentiment shared by most working in emergency departments globally (WHO 2003). Documentation of key symptoms and signs that aid diagnosis of acute abdomen was also poor, and investigations were sparsely used, including microbiological investigations.

A relatively small proportion of admissions ultimately received surgery, while the majority of long‐bone fractures and burns were treated conservatively. In the limited number of cases that ultimately required an operation, the rationale was not always well documented. Pre‐operative assessment of vital signs was inconsistently recorded, signed consent was on occasions not found in the record, and records of pre‐medication used, estimated blood loss or instrument/sponge counts were rare. Anaesthesiologist identification, which anaesthetic agents were used, and the patient condition at the end of surgery were available in about half to two‐thirds of cases. Such findings suggest that there is room for significant improvement in documentation of care provided by all members of the operative team. While tools such as checklists are available to support improved care (Soar *et al*. 2009), these were not found in the hospitals surveyed although work on essential obstetric (Hussein *et al*. 2012; Dumont *et al*. 2013) and inpatient paediatric care (Ayieko *et al*. 2011; English *et al*. 2011) suggests that improvement of services is possible with appropriately designed interventions.

Although they cannot be directly linked to patient outcomes, our findings on the structure and process of care suggest that the Kenyan Ministry of Health should devote increased attention to the quality of surgical services and the quality of experiential training being received by young doctors. In other work, we have found that efforts to monitor outcomes of hospital care through the routine health information system are undermined by poor data quality (Kihuba *et al*. 2014). A need exists for clearly defined standards for surgical care, improved access to guidelines, and monitoring and evaluation of core services against agreed standards as part of increasingly system‐wide efforts to improve care. This would be facilitated by improved routine information systems that are now a global priority (Kim 2012). While awaiting this, we have demonstrated that surveys can be useful for facilitating national and global debate on the urgent need to improve the quality and safety of surgery.
